# Use of water and ethanol extracts from wine grape seed pomace to prepare an antioxidant toothpaste

**DOI:** 10.1002/jsfa.11232

**Published:** 2021-05-03

**Authors:** Emanuela Emmulo, Brunella Ceccantoni, Andrea Bellincontro, Fabio Mencarelli

**Affiliations:** ^1^ DIBAF University of Tuscia Viterbo Italy; ^2^ DAFE University of Pisa Pisa Italy

**Keywords:** toothpaste, grape pomace, ethanol, water, solvent, polyphenols

## Abstract

**BACKGROUND:**

Extracts of fresh wine grape seeds/skin or of grape pomace seeds were used to prepare antioxidant natural toothpastes.

**RESULTS:**

Ethanol extracted twice more polyphenols than water; ultrasound did not provide any improvement in the extraction. The addition of freeze‐dried ethanol extracts of seeds or skin, at 2% and 10%, to the commercial toothpaste significantly increased the polyphenol content, both from white grape seeds and skin and from red grape seed pomace. The evaluation of time stability (shelf life) revealed a decrease, after 4 months, of 3.9% and 9.4% in total polyphenol content, in 5% and 10% water extracts, but not for ethanol extracts. 1,1‐Diphenyl‐2‐picrilhydrazil^1^ antiradical activity was the highest in 10% of seed water extract toothpaste and, after 4 months, the activity was stable.

**CONCLUSION:**

Ethanol and water are efficient and safe solvents to create natural toothpaste with grape or pomace seed extract with antioxidant activity. © 2021 The Authors. *Journal of The Science of Food and Agriculture* published by John Wiley & Sons Ltd on behalf of Society of Chemical Industry.

## INTRODUCTION

Caries and periodontitis are common challenges of our societies worldwide.^1^, ^2^ Periodontal diseases are a group of disorders characterized by an inflammatory reaction of periodontium, induced by bacterial challenge, leading to gingival inflammation, periodontal tissue destruction and alveolar bone loss.^3^ In fact, periodontal diseases occur when pathogenic microbial plaque interacts with a susceptible host,^4^ and can be prevented mainly by tooth brushing in combination with toothpaste as well as a healthy diet (e.g. low sugar intake, no excessive use of erosive drinks) and lifestyle (e.g. no smoking, low levels of stress, not being overweight).^5^, ^6^ Modern toothpastes contain many different agents for the prevention of caries and periodontitis, e.g. fluorides (sodium fluoride, amine fluoride, etc.), chlorhexidine, stannous and zinc salts, calcium phosphates such as hydroxyapatite or amorphous calcium phosphates, and surfactants as well as different abrasives for efficient plaque removal.^7^ Reactive oxygen molecules are implicated as pathological mediators in many clinical disorders,^8^ and periodontal tissue damage is related to oxidative stress due to host microbial interactions.^9^ Against oxidative damage, living organisms use an antioxidant system consisting of enzymes to convert reactive oxygen species into non‐toxic compounds and antioxidant compounds such as β‐carotene, retinol, ascorbic acid, α‐tocopherol and selenium.^10^ In the last decade, special attention by researchers has been paid to the use of plant compounds as an antimicrobial component of toothpaste, but in China the use of herbal extracts in toothpaste is common.^11^, ^12^ Most herbal extracts contain flavonoids, tannins and alkaloids. Different flavonoids (apigenin, catechin, luteolin, morin, myricetin, naringin, quercetin and rutin) have been tested against dental film bacteria and *Candida albicans* to prevent candidiasis, through the inhibition of their growth.^13^ Polyphenols of coffee, cocoa and tea beverages have been seen to prevent the cariogenic process.^14^ Use of specific flavonoids, such as (−)‐epigallocatechin‐3‐gallate in high concentration in tea, maintained the periodontal ligament cell viability of avulsed teeth.^15^ Also, the effect of proanthocyanidin (PA)‐rich grape seed extract on the biodegradation resistance of demineralized root dentine and on the bond strength and durability between resin‐based sealer and root dentine was investigated, providing interesting results in increasing the resistance of root dentine and bond strength.^16^

The recovery of antioxidant compounds from grape pomace after the vinification process is a research activity that has developed in the last decade, and several applications in medicine have been carried out to control hyperglycemia in diabetic mice^17^ or to control oxidative stress in obese mice,^18^ to protect endothelial and muscle cells through an increase in glutathione (GSH) levels as potent scavengers of free radicals,^19^ and providing protection against colon carcinogenesis.^20^ For dental care a recent review by Delimont and Carlson^21^ reported that grape seed extract properties facilitate dental caries prevention, by inhibiting proliferation of bacterial biofilms on tooth surfaces and promoting dental remineralization.^22^

In this research, the general hypothesis has been whether the potential use of seed polyphenols, mainly tannins, extracted from grape pomace after the vinification process, in a commercial toothpaste formulation could enhance its antioxidant activity by maintaining stability at room temperature. Moreover, an easy‐to‐use and low‐cost extraction method has been studied and is reported here.

## MATERIALS AND METHODS

### Matrices

Two matrices were used: (1) fresh grape of white wine grape variety Grechetto (*Vitis vinifera* L.), collecting skin and seeds; (2) pomace of variety Cabernet Sauvignon^2^ after vinification in Airmix® (Parsec srl, Florence, Italy) fermenter, collecting seeds.

### Extraction tests

Fresh grapes of white wine grape variety Grechetto were treated as follows:

1. 50 g skin and 50 g seeds of Grechetto berries were collected and added, separately, with 100 mL distilled water or 60% (v/v) ethanol (96° Reagent Pure Erba (RPE), Carlo Erba, Milan, Italy), homogenized with an UltraTurrax T25 at 30 618 × *g* for 3 min or by using an ultrasound bath for 5 min; the extracts were then centrifuged at 4 °C, 21 074 × *g* for 15 min. The supernatants were collected and analyzed by high‐performance liquid chromatography (HPLC).
2. 50 g skin and 50 g seeds of Grechetto berries were collected and, separately, ground using an IKA A11 mill (IKA‐Lab, Milan, Italy) for 10 min under liquid nitrogen. The ground samples were added to 150 mL ethanol (96° RPE, Carlo Erba, Milan, Italy), and left under stirring in the dark, for 1 h at 20 °C, then centrifuged at 4 °C at 21 074 *g* for 15 min. The supernatants were recovered, partially concentrated under vacuum at 4°C in a Rotavapor, then freeze‐dried and stored for the successive analyses.

Seeds from Airmix® (Parsec srl, Florence, Italy) vinification process of Cabernet Sauvignon variety were treated as follows. The seeds were frozen under liquid nitrogen and finely ground using an IKA A11 mill. 200 g grape seed powder was placed in a 2 L glass bottle, added to distilled water or 60% (v/v) ethanol (96° RPE, Carlo Erba, Milan, Italy), in a ratio of 1:10, and left to extract under dark conditions, at 20 °C for 1 h. The extracts were then centrifuged at 4 °C, at 21 074 × *g* for 15 min. The recovered supernatants were partially concentrated under vacuum at 4 °C in a Rotavapor, then freeze‐dried and stored for analyses. All the extractions were run in triplicate.

### Analyses

The aqueous and ethanolic extracts were characterized by reversed‐phase liquid chromatography using a Dionex chromatograph (Dionex Corporation, Sunnyvale, CA, USA), equipped with P680 quaternary pump, manual injector (Rheodyne) with 20 μL loop, TCC‐100 thermostated oven, PDA 100 detector (photodiode array detector) and controlled using Chromeleon software (version 6.50). The separation was carried out with a Dionex Acclaim® 120 C18 column, 5 μm, 4.6 × 250 mm thermostated at 30 ° C. The mobile phase consisted of a ternary gradient: solvent A = 50 mmol L^−1^ ammonium dihydrogen phosphate (99% purity) adjusted to pH 2.6 with phosphoric acid (≥85% purity); solvent B = 20% solvent A and 80% acetonitrile (99.9% purity); solvent C = 0.2 mol L^−1^ orthophosphoric acid (85% purity) adjusted to pH 1.5 with NaOH (99.99% purity). All the HPLC reagents were supplied by the Sigma‐Aldrich Chemical Co. The phenolic compounds were identified based on their elution order, the retention times of pure compounds and the characteristics of their UV–visible spectra at wavelengths of 280 nm for catechins and benzoic acids, 316 nm for hydrocinnamates, 365 nm for flavonols and 520 nm for anthocyanins.

Total polyphenol analysis was carried out by the Folin–Ciocalteu method, expressing data as concentration of gallic acid equivalents. The analysis of proanthocyanidins (PA) was carried out following the method reported by Di Stefano *et al*.^23^ The anti‐free radical activity against the stable radical DPPH was assessed by measuring the change in absorbance at 515 nm after 180 min of reaction (time needed to reach the stationary phase) carried out under dark conditions at 25 °C, according to the method reported by Bondet *et al*.,^24^ slightly modified. DPPH (0.06 mmol L^−1^) was prepared daily by diluting 1:10 a solution containing 0.0118 g DPPH in 50 mL MeOH. The DPPH concentration was corrected appropriately in order to have the initial absorbance at 515 nm of 3 mL DPPH added to 400 mL MeOH between 0.716 and 0.720. The exact concentration of DPPH in the cuvette was obtained based on the molar absorptivity value reported in the literature (12.5 mmol L^−1^ cm^−1^).^25^ The test was run by mixing 3 mL of the DPPH solution and 0.4 mL of extract in a cuvette, at increasing concentrations, diluted in MeOH. The cuvettes were capped and, after 180 min of reaction at room temperature, the absorbance was recorded at 515 nm against pure MeOH. The control was prepared using 0.4 mL methanol instead of the sample. The anti‐free radical activity was expressed as IC50 (efficient concentration), which represents the concentration of sample in the cuvette (micrograms of extract per milligram of DPPH or micrograms of advanced glycation end‐product (AGE) per milligram of DPPH) necessary to reduce the initial amount of DPPH by 50% radical. In the case of the reference antioxidants, the IC50 value was expressed in micrograms of compound per milligram of DPPH. The antiradical efficiency (EA) was calculated as the inverse of IC50.

The antiradical activity of the extracts was also expressed in terms of equivalent standard antioxidant capacity (CASE), defined as the quantity (μg) of reference antioxidant (butylated hydroxyanisole, butylated hydroxytoluene, Trolox and α‐tocopherol) having the same antiradical capacity (50% of DPPH scavenging) of a microgram of sample.

### Preparation of functional toothpaste

A commercial toothpaste characterized by absence of polyphenols, as validated by our laboratory analysis, has been added to different percentages of water or ethanolic (60% v/v) extracts of skin and seeds, extracted as described above, as follow:

- white grape skin: 2% or 10% ethanol extract;
- white grape seeds: 2% or 10% ethanol extract;
- red grape seeds: 2.5% or 5% ethanol extract;
- red grape seeds: 5% or 10% water extract.

The functional toothpaste was made by mixing the freeze‐dried extract in the appropriate percentages to the commercial toothpaste, using a spatula, to obtain a homogeneous paste, which was placed inside Eppendorf tubes, stored in the dark at room temperature (20 ± 2 °C).

To test the content of total polyphenols and the antioxidant activity of the different formulations, the samples were previously frozen and freeze‐dried and then analyzed as described above. Moreover, the shelf life of these toothpastes was monitored after 2 and 4 months at ambient temperature, measuring total polyphenols and antioxidant activity.

Finally, the functional prepared toothpastes were compared with functional commercial toothpaste (tea tree oil, gel dentifrice végétal, pâte dentifrice au ratanhia), which were analyzed for polyphenol content.

### Acceptability test of functional formulations

After the preliminary tests, we decided to concentrate the preparation of toothpastes only with the seed extracts of Cabernet Sauvignon by using selected percentages of extracts. The reason was that, by using the Airmix® vinification process, we obtained a large amount of seeds that were easy to remove, while the skin residues were very thin and completely extracted, which is the power of this technique. The different experimental formulations were offered to a group of ten untrained consumers. A 9‐point hedonistic scale was used to measure the preference and acceptability of the functional toothpastes.

The samples were presented one at a time as follows:

- toothpaste containing 5% aqueous grape seed extract;
- toothpaste containing 10% aqueous grape seed extract;
- toothpaste containing 2.5% ethanol grape seed extract;
- toothpaste containing 5% ethanol grape seed extract.

Each consumer was asked to express their hedonistic evaluation on the scale. The consumer group was also asked to report whether the formulations presented abnormal characteristics compared to common toothpastes in terms of consistency, flavor, smell and astringency.

### Statistical analysis

Analysis of variance was performed for the study of data variance, and the statistical significance of any differences was calculated by Tukey's *b*‐test at *P* < 0.05. Calculations were performed in Minitab 15 (Minitab, Inc., State College, PA, USA).

## RESULTS AND DISCUSSION

HPLC analysis, conducted on the extracts of fresh white grapes in the preliminary tests, highlighted the extraction efficiency of absolute ethanol; regarding flavonol compounds we concentrated as a target on quercetin (3,5,7,3′,4′‐pentahydroxyflavone), which is the main representative of the flavonol subclass in grape berry, and it has been shown to have a broad variety of biological activities and pharmacological actions; these properties are largely attributed to quercetin's ability to diminish the formation of reactive oxygen species (ROS) through different mechanisms.^26^ Quercetin concentration in ethanol was about double than in the extracts from water solvent, confirming the observation by Pasini *et al*.^27^ The use of ultrasound did not provide any improvement in extraction capacity (Table 1). This last finding is in contrast to what is reported in the literature.^28^ Moisture content of the sample, milling degree, particle size and solvent are very important factors in obtaining efficient and effective extraction. Furthermore, temperature, pressure, frequency and time of sonication are the governing factors for the action of ultrasound. Too many variables can affect the performance. Moreover, the effect of solvent (ethanol) is more important than that of ultrasound; indeed, Rostagno *et al*.^29^ found that ultrasound can improve the extraction yield, depending on solvent use.

**Table 1:** Extraction methods evaluated by HPLC on the basis of quercetin concentration (area value in mAu)

Sample	Quercetin glucoside (mg L^−1^)
Ethanol/extract 1/4	20.6 ± 2.2a
Ultrasounds + ethanol/extract 1/4	19.7 ± 1.6a
Water/extract 1/4	10.4 ± 1.2b
Ultrasound + water/extract 1/4	12.7 ± 1.0b

Data are the mean of five extracts from different skin samples (±SD). In the column, different letters indicate significance at *P* < 0.05.

The total polyphenol content in the extracts of seeds and skin from fresh white grape is reported in Table 2 and compared to that of commercial toothpaste used for the extract addition. The total polyphenol content in commercial toothpaste is almost 0, which is why we used this commercial toothpaste as a basis for extract addition. As expected, the seeds had a higher (almost double) amount of polyphenols than the skin, being white grape berries.

**Table 2:** Total polyphenol content of commercial toothpaste, white grape ethanol (60% v/v) extracts and commercial toothpaste with addition of ethanol extracts

Sample	Gallic acid equivalents (mg g^−1^ of extract)
Commercial toothpaste	3.6 ± 0.3e
Seed extract	521.1 ± 10.6a
Skin extract	285.7 ± 10.2c
Commercial toothpaste + 2% seed extract	4.3 ± 0.3de
Commercial toothpaste + 2% skin extract	3.9 ± 0.1de
Commercial toothpaste + 10% seed extract	28.1 ± 1.9b
Commercial toothpaste + 10% skin extract	4.9 ± 1.4d

Data are the mean of five extracts from different seeds and skin samples (±SD). In the column, different letters indicate significance at *p* < 0.05.

The addition of 2% or 10% freeze‐dried ethanol extracts of seeds or skin to the commercial toothpaste significantly increased the polyphenol content in the commercial toothpaste + seed extract 10% sample, compared to that with 2% of extract, whereas the difference was not significant between 2% and 10% for the skin extract samples (Table 2). This last result can probably be attributed to the rapid oxidability of the extract when it is in low concentration in the toothpaste.

In the second part of the research, temporally speaking, Cabernet Sauvignon pomace (mainly seeds) after the Airmix® vinification process was analyzed for total polyphenol content in water and ethanol extracts (Table 3). The much lower content in Cabernet seed extract than that in white grape skin and seed extracts is mainly due to most polyphenols having been extracted during vinification. However, the strong ability of ethanol to extract polyphenols – almost sevenfold more than water – was confirmed. The concentrations were slightly higher than expected, as percentages of added extracts, due to the rapid evaporation of water from the paste during the analysis procedure. The functional commercial toothpaste (tea tree oil, gel dentifrice végétal, pâte dentifrice au ratanhia) did not show any polyphenol content; this seems strange because these toothpastes contain herb extracts generally known for their high content of polyphenols. It is likely that the drying method and/or storage conditions contributed to phenol loss.

**Table 3:** Total polyphenol content in seed water and ethanol (60% v/v) extracts of Cabernet Sauvignon, in commercial toothpaste with addition of 10% extract, and in commercial functional toothpastes

Sample	Gallic acid equivalents (mg g^−1^ of extract)
Seed water extract	74.5 ± 6.4b
Seed ethanol extract	263.9 ± 26.3a
Commercial toothpaste + 5% seed water extract	4.5 ± 0.4f
Commercial toothpaste + 10% seed water extract	9.2 ± 1.1e
Commercial toothpaste + 2.5% seed ethanol extract	20.1 ± 3.2d
Commercial toothpaste + 5% seed ethanol extract	30.7 ± 4.0c
Tea tree oil	Trace
Gel dentifrice végétal	Trace
Pâte dentifrice au ratanhia	Trace

Data are the mean of five extracts from different skin or seed samples (±SD). In the column, different letters indicate significance at *P* < 0.05.

The same procedure used for white grape skin and seed extract was followed for Cabernet Sauvignon seed extract, but using freeze‐dried water extract and ethanol extract to add to the commercial toothpaste. The evaluation of time stability (shelf life; Table 4) revealed a 3.9% and 9.4% decrease in total polyphenol content, after 2 months, respectively, in 5% and 10% water extracts; this loss persisted after 4 months. In contrast, the ethanol extract toothpastes showed a 3.5% and 3.7% increase after 2 months, which remained after 4 months; this light concentration effect for the ethanol extract samples is due to the greater evaporation coefficient of ethanol than water. With regard to antiradical activity, the lowest value, and thus the highest antioxidant power, was found in the samples with 10% of seed water extract and remained the lowest also during storage (Table 4). The lowest antioxidant activity was for the samples with the lowest amount of seed extract both for water and for ethanol. However, after 4 months, the antioxidant activities of the 10% seed water extract and 5% seed ethanol extract were similar, meaning that ethanol had a better preservation action than water. The highest anti‐free radical activity against the stable radical DPPH, shown by the sample with 10% of seed water extract, was unexpected because the total polyphenol content in seed water extract was significantly lower than that in seed ethanol extract, as shown in Table 3, and also polyphenols showed reduced concentrations after 2 and 4 months (Table 4). We assume that this could be due to the antioxidant effect of different classes of polyphenols but, overall, the result of extraction of the seed lipid fraction by ethanol. In grape seeds the most concentrated class is flavanols, proanthocyanidins or condensed tannins, which have a strong antioxidant activity and have been studied for their important role in dental caries prevention.^21^ Depending on the solvent used or on the method of extraction, lipids are more or less intensively extracted and it is known that oil has very moderate antiradical activity, as determined by DPPH radical scavenging, in contrast to the strong antiradical activity of polyphenols; but, overall, the lipid fraction is easily oxidizable, producing a radical which increases the oxidability of the substrate.^30^

**Table 4:** Changes in total polyphenol content after 2 and 4 months of toothpaste storage at room temperature, and antioxidant activity (micrograms of advanced glycation end‐product (AGE) per milligram of DDPH) at the time of preparation and after 2 and 4 months at room temperature

	Change in polyphenols (%)		μg AGE mg^−1^ DDPH		
	After 2 months	After 4 months	Initial	After 2 months	After 4 months
Commercial toothpaste + 5% seed water extract	−3.9	−3.9	317.8 ± 10.3d	383.6 ± 10.6b	561.5 ± 20.2a
Commercial toothpaste + 10% seed water extract	−9.4	−7.5	162.7 ± 7.0 g	163.6 ± 7.9 g	261.3 ± 10.0e
Commercial toothpaste + 2.5% seed ethanol extract	3.7	3.7	347.0 ± 15.0c	355.2 ± 11.2bc	564.5 ± 20.6a
Commercial toothpaste + 5% seed ethanol extract	3.5	3.5	198.3 ± 16.0f	221.3 ± 11.2f	288.8 ± 13.1e

Data are the mean of five extracts from different seed samples (±SD). In each column of antioxidant activity (μg AGE mg^−1^ DDPH), different letters indicate significance at *P* < 0.05.

Most of the samples were judged as ‘neither pleasant nor unpleasant’; this is probably due to the fact that, as expressed by many, the flavor of the extracts was masked by the mint flavor of the commercial toothpaste base. The toothpaste with 10% seed water extract was considered by three judges to be ‘slightly unpleasant’. The observations reported by two of these three judges were: ‘the toothpaste has a bitter taste’ and ‘toothpaste reminds me of wet grass.’ Toothpastes with seed ethanol extracts had almost the same level of appreciation. In contrast, the most appreciated toothpaste was the one with 5% seed water extract. This can be attributed to this sample having the lowest concentration of proanthocyanidins; indeed, in seeds, they are less polymerized than in skins, greatly affecting the astringency and acceptability of the wine (Table 5).^31^

**Table 5:** Acceptability tests of the experimental toothpastes. Data report the number of untrained tasters who expressed the acceptability terms of the first column

	Commercial toothpaste + 5% seed water extract	Commercial toothpaste + 10% seed water extract	Commercial toothpaste + 2.5% seed ethanol extract	Commercial toothpaste + 5% seed ethanol extract
Pleasant	3	0	1	1
Slightly pleasant	3	1	1	2
Neither pleasant nor unpleasant	4	6	8	7
Slightly unpleasant	0	3	0	0
Unpleasant	0	0	0	0

## CONCLUSIONS

White or red grape pomace extracts can be used to produce stable toothpastes at room temperature which have a significant antioxidant activity. Ethanol is the best solvent but also water could be used; under our conditions the use of an ultrasonic bath did not show any improvement in extraction. In terms of consumer appreciation at a laboratory sensory test, the commercial toothpaste + 5% seed water extract was considered pleasant or slightly pleasant. An easy, low‐cost, and sustainable method to produce toothpaste by valorizing vinification byproducts is proposed.

## Footnotes

2 …Cabernet Sauvignon (*Vitis vinifera* L.)

## References

[1] Fejerskov O , Nyvad B, Kidd E. Dental Caries: The Disease and its Clinical Management, 3rd edn. Wiley‐Blackwell (2015) 480 p.

[2] Meyer F , Karch A , Schlinkmann KM , Dreesman J , Horn J , Rübsamen N *et al*., Sociodemographic determinants of spatial disparities in early childhood caries: an ecological analysis in Braunschweig Germany. Community Dent Oral Epidemiol 45:442–448 (2017). 28547864 10.1111/cdoe.12308

[3] Haffajee AD , Microbial etiological agents of destructive periodontal diseases. Periodontology 2000 5:78–111 (1994). 9673164 10.1111/j.1600-0757.1994.tb00020.x

[4] Van Dyke TE , Lester MA and Shapira L , The role of the host response in periodontal disease progression: implications for future treatment strategies. J Periodontol 64:792–806 (1993). 10.1902/jop.1993.64.8s.792 8410619

[5] van Loveren C , Toothpastes. Monogr Oral Sci. S. Karger AG, Basel, Switzerland, (2013) vol. 23, 158 p.

[6] Yaacob M , Worthington HV , Deacon SA , Deery C , Walmsley AD , Robinson PG *et al*., Powered versus manual toothbrushing for oral health, in Cochrane Database of Systematic Reviews. Wiley, Chichester (2014). https://www.ncbi.nlm.nih.gov/pmc/articles/PMC7133541/ 10.1002/14651858.CD002281.pub3 PMC7133541 24934383

[7] Epple M , Meyer F and Enax J , A critical review of modern concepts for teeth whitening. Dent J 7:79 (2019). 10.3390/dj7030079 PMC6784469 31374877

[8] Slater TF , Cheeseman KH , Davies MJ , Proudfoot K and Xin W , Free radical mechanisms in relation to tissue injury. Proc Nutr Soc 46:1–12 (1987). 3033678 10.1079/pns19870003

[9] Pendyala G , Thomas B and Kumari S , The challenge of antioxidants to free radicals in periodontitis. J Indian Soc Periodontol 12:79–83 (2008). 10.4103/0972-124X.44100 PMC2813562 20142950

[10] Asman B , Wijkander P and Hjerpe A , Reduction of collagen degradation in experimental granulation tissue by vitamin E and selenium. J Clin Periodontol 21:45–47 (1994). 8126243 10.1111/j.1600-051x.1994.tb00275.x

[11] Hotwani K , Baliga S and Sharma K , Phytodentistry: use of medicinal plants. J Complementary Integr Med 11:233–251 (2014). 10.1515/jcim-2013-0015 25153610

[12] Giraudi M , Romano F and Aimetti M , An update on herbal anti‐inflammatory agents in periodontal therapy. Clin Anti‐Inflammatory Anti‐Allergy 2:27–37 (2015).

[13] Gutiérrez‐Venegas G , Gómez‐Mora JA , Meraz‐Rodríguez MA , Flores‐Sánchez MA and Ortiz‐Miranda LF , Effect of flavonoids on antimicrobial activity of microorganisms present in dental plaque. Heliyon 5:e03013 (2019). 31886429 10.1016/j.heliyon.2019.e03013 PMC6921118

[14] Ferrazzano GF , Amato I , Ingenito A , De Natale A and Pollio A , Anti‐cariogenic effects of polyphenols from plant stimulant beverages (cocoa, coffee, tea). Fitoterapia 80:255–262 (2009). 19397954 10.1016/j.fitote.2009.04.006

[15] Jung I‐H , Yun J‐H , Cho A‐R , Kim C‐S , Chung W‐G and Choi S‐H , Effect of (−)‐epigallocatechin‐3‐gallate on maintaining the periodontal ligament cell viability of avulsed teeth: a preliminary study. J Periodontal Implant Sci 41:10–16 (2011). 21394292 10.5051/jpis.2011.41.1.10 PMC3051051

[16] Kalra M , Iqbal K , Nitisusanta LI , Daood U , Sum CP and Fawzy AS , The effect of proanthocyanidins on the bond strength and durability of resin sealer to root dentine. Int Endod J 46:169–178 (2013). 22900674 10.1111/j.1365-2591.2012.02106.x

[17] Hogan S , Zhang L , Li J , Sun S , Canning C and Zhou K , Antioxidant rich grape pomace extract suppresses postprandial hyperglycemia in diabetic mice by specifically inhibiting alpha‐glucosidase. Nutr Metab 7:71 (2010). 10.1186/1743-7075-7-71 PMC2939653 20799969

[18] Hogan S , Canning C , Sun S , Sun X and Zhou K , Effects of grape pomace antioxidant extract on oxidative stress and inflammation in diet induced obese mice. J Agric Food Chem 58:11250–11256 (2010). 20929236 10.1021/jf102759e

[19] Goutzourelas N , Stagos D , Housmekeridou A , Karapouliou C , Kerasioti E , Aligiannis N *et al*., Grape pomace extract exerts antioxidant effects through an increase in GCS levels and GST activity in muscle and endothelial cells. Int J Mol Med 36:433–441 (2015). 26082074 10.3892/ijmm.2015.2246 PMC4501638

[20] Tian Q , Xu Z , Sun X , Deavila J , Du M and Zhu M , Grape pomace inhibits colon carcinogenesis by suppressing cell proliferation and inducing epigenetic modifications. J Nutr Biochem 84:108443 (2020). 10.1016/j.jnutbio 32629240

[21] Delimont NM and Carlson BN , Prevention of dental caries by grape seed extract supplementation: a systematic review. Nutr Health 26:43–52 (2020). 31760860 10.1177/0260106019887890

[22] Nagi SM , Hassan SN , Abd El‐Alim SH and Elmissiry MM , Remineralization potential of grape seed extract hydrogels on bleached enamel compared to fluoride gel: an in vitro study. J Clin Exp Dent 11:e401 (2019). 31275511 10.4317/jced.55556 PMC6599700

[23] Di Stefano R , Cravero MC and Gentilini N , Metodi per lo studio dei polifenoli dei vini. L'Enotecnico 25:83–89 (1989).

[24] Bondet V , Brand‐Williams W and Berset C , Kinetics and mechanisms of antioxidant activity using the DPPH∙ free radical method. Food Sci Technol 30:609–615 (1997).

[25] Arnao MB , Some methodological problems in the determination of antioxidant activity using chromogen radicals: a practical case. Trends Food Sci Technol 11:419–421 (2000).

[26] Vasquez‐Espinal A , Yanez O , Osorio E , Areche C , Garcia‐Beltran O , Ruiz LM *et al*., Theoretical study of the antioxidant activity of quercetin oxidation products. Front Chem 7:818 (2019). 31828060 10.3389/fchem.2019.00818 PMC6890856

[27] Pasini F , Chinnici F , Caboni MF and Verardo V , Recovery of oligomeric proanthocyanidins and other phenolic compounds with established bioactivity from grape seed by‐products. Molecules 24:677 (2019). 10.3390/molecules24040677 PMC6413075 30769803

[28] Azmir J , Zaidul ISM , Rahman MM , Sharif KM , Mohamed A , Sahena F *et al*., Techniques for extraction of bioactive compounds from plant materials: a review. J Food Eng 117:426–436 (2013).

[29] Rostagno MA , Palma M and Barroso CG , Pressurized liquid extraction of isoflavones from soybeans. Anal Chim Acta 522:169–177 (2004).

[30] Johnson DR and Decker EA , The role of oxygen in lipid oxidation reactions: a review. Annu Rev Food Sci Technol 6:171–190 (2015). 25665172 10.1146/annurev-food-022814-015532

[31] Castellarin SD , Bavaresco L , Falginella L , MVZ G and Di Gaspero G , Phenolics in Grape Berry and Key Antioxidants. Bentham Science, Sharjah, UAE (2012).

